# Deep Learning-Based Estimation of Myocardial Material Parameters from Cardiac MRI

**DOI:** 10.3390/bioengineering12040433

**Published:** 2025-04-21

**Authors:** Yunhe Chen, Xiwen Zhang, Yongzhong Huo, Shuo Wang

**Affiliations:** 1 Department of Aeronautics and Astronautics, Fudan University, Shanghai 200433, China; yunhechen23@m.fudan.edu.cn; 2 Digital Medical Research Center, School of Basic Medical Sciences, Fudan University, Shanghai 200032, China; 20307110438@fudan.edu.cn; 3 Shanghai Key Laboratory of Medical Imaging Computing and Computer Assisted Intervention, Shanghai 200032, China

**Keywords:** myocardial material parameters, deep learning, cardiac magnetic resonance imaging, finite element method

## Abstract

Background: Accurate estimation of myocardial material parameters is crucial to understand cardiac biomechanics and plays a key role in advancing computational modeling and clinical applications. Traditional inverse finite element (FE) methods rely on iterative optimization to infer these parameters, which is computationally expensive and time-consuming, limiting their clinical applicability. Methods: This study proposes a deep learning-based approach to rapidly and accurately estimate the left ventricular myocardial material parameters directly from routine cardiac magnetic resonance imaging (CMRI) data. A ResNet18-based model was trained on FEM-derived parameters from a dataset of 1288 healthy subjects. Results: The proposed model demonstrated high predictive accuracy on healthy subjects, achieving mean absolute errors of 0.0146 for $C_{a}$ and 0.0139 for $C_{b}$, with mean relative errors below 5.00%. Additionally, we evaluated the model on a small pathological subset (including ARV and HCM cases). The results revealed that while the model maintained strong performance on healthy data, the prediction errors in the pathological samples were higher, indicating increased challenges in modeling diseased myocardial tissue. Conclusion: This study establishes a computationally efficient and accurate deep learning framework for estimating myocardial material parameters, eliminating the need for time-consuming iterative FE optimization. While the model shows promising performance on healthy subjects, further validation and refinement are required to address its limitations in pathological conditions, thereby paving the way for personalized cardiac modeling and improved clinical decision-making.

## 1. Introduction

Myocardial material parameters are key indicators of maladaptive cardiac remodeling and are closely linked to the pathogenesis of various heart diseases [1]. As critical biomarkers of overall cardiac function, accurate assessment of these parameters facilitates the early and precise diagnosis of structural heart diseases and supports individualized treatment, ultimately reducing the incidence and mortality of cardiac conditions [2]. Over the past few decades, significant progress has been made in cardiac mathematical modeling and numerical simulation [3]. Myocardial tissue is typically regarded as a hyperelastic material with pronounced nonlinear anisotropic stress responses. To characterize myocardial material’s behavior, various constitutive models have been proposed; notably, the Holzapfel–Ogden (HO) model [4]—a structure-based orthotropic model—has been widely adopted.

In recent years, advances in medical imaging techniques [5], particularly magnetic resonance imaging (MRI) [6,7], have enabled the accurate extraction of cardiac geometries, thereby providing essential geometric data for patient-specific inverse finite element (FE) estimation of myocardial material parameters [8]. However, conventional inverse FE methods often require repeated iterations to solve complex nonlinear optimization problems that minimize the discrepancy between the measured end-diastolic pressure–volume relationship (EDPVR) and the FE model predictions [9,10]. This iterative process not only incurs high computational costs but is also time-consuming, making it unsuitable for real-time clinical diagnosis.

Recently, to reduce the computational cost associated with constrained optimization in the inverse estimation of material parameters using traditional FEM in cardiac models, researchers have adopted machine learning surrogate models as an alternative to conventional FEM. This approach enables rapid parameter inference directly from imaging and other data, providing an efficient and convenient solution for biomechanical modeling. For instance, Liang et al. [11] developed a deep learning model capable of directly estimating the stress distribution in the aorta. The model not only exhibits exceptional predictive accuracy but also operates at an extremely fast speed. This study was the first to demonstrate the tremendous potential of using deep learning techniques to replace traditional finite element analysis (FEA) in stress analysis. Dabiri et al. [12] employed eXtreme Gradient Boosting (XGBoost) and Cubist algorithms to predict the LV pressure, volume, and stress. The experimental results demonstrated that the ML surrogate models can rapidly and accurately predict the LV mechanics. Cai et al. [13] developed three machine learning surrogate models that accurately capture the intrinsic relationships between the pressure–volume and pressure–strain, thereby enabling the rapid estimation of the passive parameters of the left ventricular myocardium. The three aforementioned studies primarily explored the feasibility and reliability of using machine learning surrogate models to replace conventional forward finite element simulations for efficiently and accurately predicting mechanical parameters. However, their applications remain relatively limited, and significant challenges persist in generalizing these surrogate models to entirely new cardiac anatomical structures. Babaei et al. [14] proposed a machine learning-based model and generated 2500 training samples using Latin hypercube sampling. The model is capable of directly predicting the passive parameters of the myocardium from the EDPVR inputs, providing an efficient and accurate tool for rapid assessment of myocardial mechanical properties. The three aforementioned studies primarily explored the feasibility and reliability of using machine learning surrogate models to replace conventional forward finite element simulations for efficiently and accurately predicting mechanical parameters. However, their applications remain relatively limited, and significant challenges persist in generalizing these surrogate models to entirely new cardiac anatomical structures. Furthermore, it is important to note that these models were predominantly trained and validated on synthetic datasets, which may limit their immediate applicability in real-world clinical settings.

In this paper, we propose an innovative deep learning-based method for the rapid estimation of myocardial material parameters directly from routine cardiac magnetic resonance imaging (CMRI) data. Unlike traditional iterative optimization methods, our approach is the first to successfully derive myocardial material parameters from a large-scale, real-world dataset, thereby substantially improving the computational efficiency while maintaining high accuracy. Specifically, we first estimated the myocardial material parameters for 1288 real CMR images using conventional inverse FE methods; subsequently, these data were employed as the training set to develop a deep neural network model capable of directly predicting myocardial material parameters from CMRI images. The experimental results demonstrate that our approach not only achieves accurate estimation of myocardial material parameters but also exhibits robust generalizability, offering an efficient new pathway for personalized cardiac modeling with significant clinical translational potential.

## 2. Materials and Methods

### 2.1. Study Population

The dataset includes 1288 high-resolution cine CMR images from healthy volunteers, all sourced from Hammersmith Hospital, Imperial College London [15]. The high-resolution cohort comprises short-axis cine CMR images captured at the end-systole (ES) and end-diastole (ED), each accompanied by detailed segmentation masks, and acquired using a 3D cine balanced steady-state free precession (b-SSFP) sequence that yields a spatial resolution of 1.25 × 1.25 × 2 mm.

### 2.2. Data Preprocessing

A validated cardiac segmentation and co-registration framework was employed, as detailed in previous studies [16,17]. Patient-specific cardiac structures were reconstructed from the bSSFP cine MRI scans. A convolutional neural network (CNN) was used to automatically segment the left ventricle and myocardium in the short-axis images.

### 2.3. Cardiac Finite Element Simulation

To create a patient-specific computational model, a tetrahedral mesh was generated from a healthy left ventricle template [17] and then aligned to each subject’s anatomy.

The passive myocardial behavior was modeled using the Holzapfel–Ogden (HO) constitutive model, with the strain energy function defined as [4]:

(1) $$\psi =\frac{a}{2b}e^{b(I_{1}-3)}+\sum_{i=f,s}\frac{a_{i}}{2b_{i}}\left[e^{b_{i}{\left(I_{4i}-1\right)}^{2}}-1\right]+\frac{a_{fs}}{2b_{fs}}[e^{b_{fs}I_{8fs}}-1]$$

where a, b, $a_{f}$, $b_{f}$, $a_{s}$, $b_{s}$, $a_{fs}$, $b_{fs}$ are eight myocardial material parameters, and $I_{1}$, $I_{4f}$, $I_{4s}$, and $I_{8fs}$ are four invariants that characterize the deformation state of myocardial tissue.

To simplify the parameter estimation, the eight HO parameters were grouped into two groups: $a^{G}=\{a,a_{f},a_{s},a_{fs}\}$ and $b^{G}=\{b,b_{f},b_{s},b_{fs}\}$ [18]. The scaling factors $C_{a}$ and $C_{b}$ were then applied to their reference values (Table 1) to determine the final material properties:

(2) $$a^{G}=C_{a}a_{0}^{G},\;b^{G}=C_{b}b_{0}^{G}$$

### 2.4. Inverse Parameter Estimation

Given the quasi-static nature of diastolic filling, the governing equilibrium equations for cardiac mechanics were formulated as follows [20]:

(3) $$\left\{\begin{array}{l} \nabla \cdot \sigma +b=0,\;\;\;\;\;\;\;\;\;\;\;\;\;\;\;\;\mathrm{in}\;\Omega \\ \sigma \cdot n=-p_{endo}n,\;\;\;\;\;\;\;\;\;\;\mathrm{in}\;\Gamma^{N}\\ u_{z}=0,\;\;\;\;\;\;\;\;\;\;\;\;\;\;\;\;\;\;\;\;\;\;\;\;\;\mathrm{in}\;\Gamma^{D} \end{array}\right.$$

where σ represents the Cauchy stress, b represents the body force density, Ω is the computational domain, n is the unit normal direction to the endocardial surface, $p_{endo}$ represents the LV filling pressure, $\Gamma^{N}$ is the Neumann boundary, $u_{z}$ represents the zero-displacement Dirichlet boundary condition on the basal plane, and $\Gamma^{D}$ is the Dirichlet boundary.

In the absence of direct LV filling pressure measurements, an 8 mmHg end-diastolic pressure was used, following Gao et al. [21].

**Figure 1:**
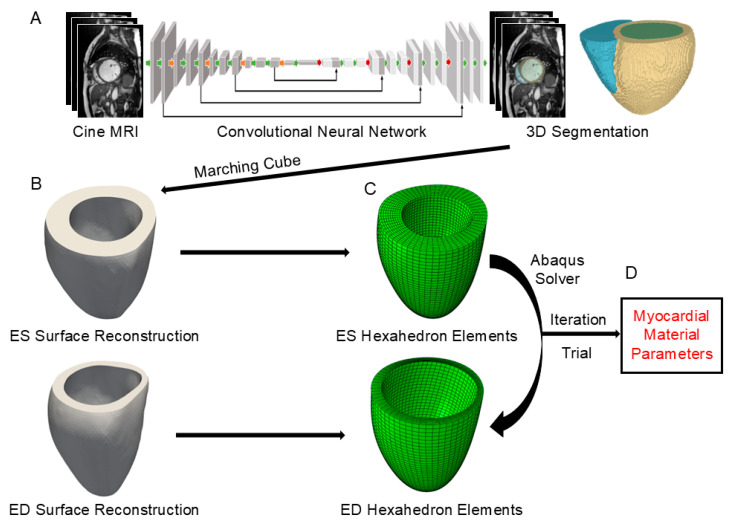
Workflow diagram for estimating myocardial material parameters using traditional inverse finite element methods. (**A**) A convolutional neural network is employed to automatically segment the cardiac structures from the short-axis cine MRI. (**B**) The resulting segmentation is used to reconstruct an enclosed surface of the left ventricular myocardium using the marching cubes algorithm. (**C**) A hexahedral mesh is generated for the myocardium. (**D**) The myocardial material parameters are estimated from the traditional inverse FE.

The LV geometry at the ES and ED was extracted, and an interactive MATLAB R2022a (MathWorks, Natick, MA, USA)–Abaqus 2022 (Dassault Systèmes, Providence, RI, USA) framework was used to estimate the material parameters (Figure 1).

### 2.5. Deep Learning Network Architecture

**Figure 2:**
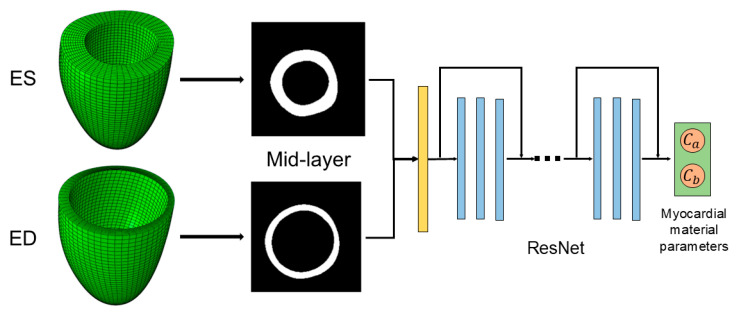
ResNet-based framework for myocardial material parameter estimation. Input: A pair of mid-ventricular short-axis slices extracted from the cine CMR at the end-systole (ES) and end-diastole (ED). ResNet architecture: The modified ResNet18 processes the two-channel input. Output: The predicted left ventricular myocardial material parameters $C_{a}$ and $C_{b}$.

In this study, two deep convolutional neural network architectures, ResNet18 and DenseNet121, were employed. Both networks were modified to accept two-channel input images by adjusting the first convolutional layer. Each input sample consisted of a pair of mid-ventricular short-axis slices extracted from the cine CMR at the end-systole (ES) and end-diastole (ED). The final fully connected layers were adapted to output two continuous values, corresponding to the estimated left ventricular material parameters $C_{a}$ and $C_{b}$. In addition, we experimented with a vision-transformer (ViT)-based model, which was similarly modified to accept two-channel inputs and to perform regression on the material parameters. Although the ViT model achieved performance metrics that were competitive with those of the CNN-based models, its training time and computational demands were significantly higher. Consequently, ResNet18 was ultimately selected as the final network model for its superior efficiency and faster convergence. The framework of ResNet is illustrated in Figure 2.

### 2.6. Network Training

Before training, the dataset was randomly split into training (70%), validation (15%), and test (15%) sets to ensure robust model evaluation. The training and validation sets were used for model optimization and hyperparameter tuning, while the test set was reserved for independent performance assessment.

Extensive hyperparameter tuning was performed by exploring the variations in several key parameters. Specifically, the Adam optimizer’s betas were set to one of [(0.9, 0.999), (0.95, 0.999), (0.9, 0.98)], the learning rates were varied over [1 × 10^−8^, 1 × 10^−7^, 1 × 10^−6^, 1 × 10^−5^, 1 × 10^−4^, 1 × 10^−3^], and the batch sizes were chosen from (16, 32, 64). Training was performed using the PyTorch framework (version 2.2.0) on a Linux platform equipped with an Intel^®^ Core™ i9-14900KF processor (Intel Corporation, Santa Clara, CA, USA) and an NVIDIA GeForce RTX 4090 GPU (NVIDIA Corporation, Santa Clara, CA, USA). The network was trained for 300 epochs with early stopping to prevent overfitting.

### 2.7. Loss Function and Performance Metrics

The mean squared error (MSE) was used as the primary loss function:

(4) $$MSE=\frac{1}{N}\sum_{i=1}^{N}{\left(y_{i}^{pred}-y_{i}^{true}\right)}^{2},$$

where N represents the total number of samples, $y_{i}^{pred}$ denotes the model’s predicted value for the ith sample, and $y_{i}^{true}$ indicates the corresponding ground truth value.

Additionally, the mean absolute error (MAE) was tracked:

(5) $$MAE=\frac{1}{N}\sum_{i=1}^{N}\left|y_{i}^{pred}-y_{i}^{true}\right|.$$

These loss metrics served as the primary indicators of model performance on the validation set. The training process involved a systematic search over the aforementioned hyperparameter space, with all the configuration results recorded for subsequent analysis.

The model performance was evaluated using several quantitative metrics, including the mean squared error (MSE), mean absolute error (MAE), and mean relative error (MRE), calculated separately for $C_{a}$ and $C_{b}$. The MRE is defined as:

(6) $$MRE=\frac{1}{N}\sum_{i=1}^{N}\frac{\left|y_{i}^{pred}-y_{i}^{true}\right|}{\left|y_{i}^{true}\right|}.$$

### 2.8. Quality Control

Data quality control was performed at two levels.

Image exclusion: Subjects with missing short-axis CMR images or poor segmentation quality were removed.

Simulation filtering: Subjects whose FEM-simulated LV volume error exceeded 0.005 or whose $C_{a}$ or $C_{b}$ values were outside the predefined upper or lower bounds were excluded. This exclusion was implemented to ensure that the data used maintained high accuracy and physiological plausibility, thereby minimizing the impact of extreme errors or outlier parameters on the model’s predictions and improving the reliability and stability of the parameter estimations.

### 2.9. Statistical Analysis

For the myocardial material parameters predicted by the model, we quantified the error relative to the ground truth values obtained from the FEM. Specifically, we reported the MSE, MAE, and MRE for each predicted parameter. Additionally, linear regression analysis was conducted to assess the correlation between the predicted and FEM-derived values, with the regression equation and Pearson correlation coefficient (r) reported.

To compare the model performance, we first assessed the normality of the validation MAE values using the Shapiro–Wilk test. For normally distributed data, a paired t-test was used, while for non-normally distributed data, the Mann–Whitney U test was applied. A *p*-value below 0.05 indicates a statistically significant difference between the models, suggesting one architecture outperforms the other.

## 3. Results

### 3.1. Distribution of FEM-Derived Myocardial Material Parameters

**Figure 3:**
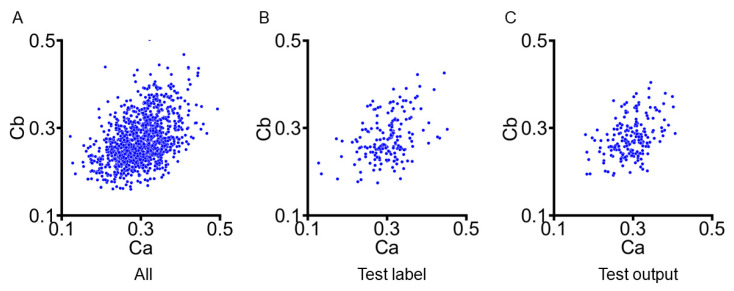
Comparison of myocardial material parameter distributions. (**A**) All the FEM-derived myocardial material parameters. (**B**) Test label. (**C**) Test output.

The FEM was used to derive reference values for the myocardial material parameters, $C_{a}$ and $C_{b}$, across the entire dataset. To provide a baseline for the model evaluation, we analyzed the distribution of these parameters within the healthy cohort. Figure 3A presents a scatterplot that visualizes the relationship between $C_{a}$ (*x*-axis) and $C_{b}$ (*y*-axis). In the healthy subjects, the FEM-derived values of $C_{a}$ and $C_{b}$ show relatively narrow distributions, which reflect the uniformity of the tissue properties in this group. These distribution patterns serve as an important reference for evaluating the performance of our deep learning model.

### 3.2. Hyperparameter Tuning Results

**Figure 4:**
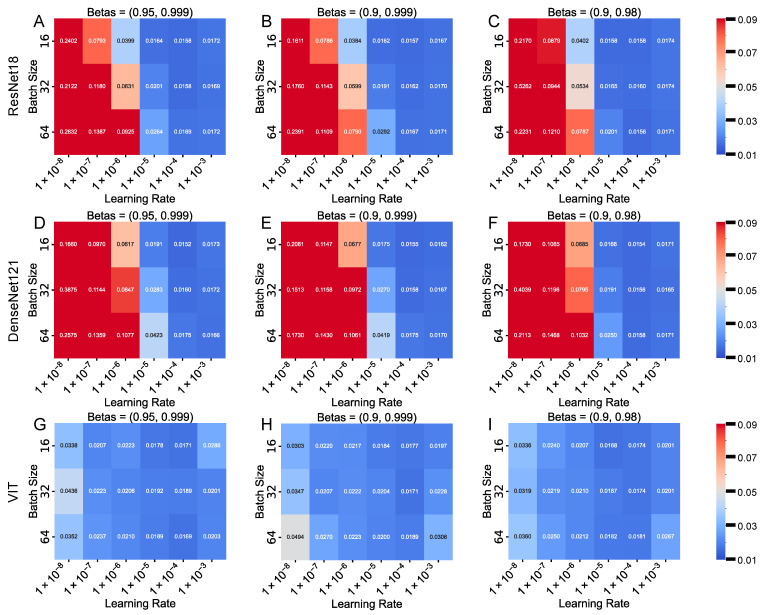
Heatmaps of the best validation MAE for hyperparameter tuning. (**A**) ResNet18, betas = (0.95, 0.999). (**B**) ResNet18, betas = (0.9, 0.999). (**C**) ResNet18, betas = (0.9, 0.98). (**D**) DenseNet121, betas = (0.95, 0.999). (**E**) DenseNet121, betas = (0.9, 0.999). (**F**) DenseNet121, betas = (0.9, 0.98). (**G**) ViT, betas = (0.95, 0.999). (**H**) ViT, betas = (0.9, 0.999). (**I**) ViT, betas = (0.9, 0.98). In each panel, the *x*-axis represents the learning rates and the *y*-axis denotes the batch sizes, with the color intensity indicating the magnitude of the best validation MAE.

We conducted hyperparameter tuning on the ResNet18, DenseNet121, and ViT architectures, exploring the effects of different Adam optimizer betas [(0.9, 0.999), (0.95, 0.999), (0.9, 0.98)], learning rates [1 × 10^−8^, 1 × 10^−7^, 1 × 10^−6^, 1 × 10^−5^, 1 × 10^−4^, 1 × 10^−3^], and batch sizes (16, 32, 64). Based on the best validation MAE, we visualized the impact of these hyperparameter choices using 2D heatmaps (Figure 4), where the *x*-axis represents the learning rate, the *y*-axis represents the batch size, and the color intensity reflects the best validation MAE. The heatmaps reveal that when the learning rate is below 1 × 10^−5^, the MAE is generally high. However, when the learning rate lies between 1 × 10^−5^ and 1 × 10^−4^, the MAE drops significantly, with smaller batch sizes more likely to yield a lower MAE.

For the healthy subject dataset, both ResNet18 and DenseNet121 demonstrated excellent predictive performance. Under optimal hyperparameter configurations, ResNet18 consistently achieved validation MAE values in the range of approximately 0.015–0.017, while DenseNet121 obtained similar results, with some configurations yielding slightly lower errors, particularly for betas = (0.95, 0.999) at a batch size of 16.

In addition, we experimented with a ViT-based model in the same hyperparameter search space. Although the ViT model achieved competitive performance—with a best validation MAE of approximately 0.0167–0.0168, which is very close to the results obtained using CNN-based architectures—its computational cost and training time were significantly higher, with the training time being more than four times that of ResNet18.

Further statistical analysis using the Mann–Whitney U test (*p* > 0.05) confirmed that the performance difference between ResNet18 and DenseNet121 was not statistically significant, suggesting that either architecture could be used interchangeably based on the accuracy alone. Given that ResNet18 required significantly less training time while maintaining a comparable level of accuracy, we ultimately adopted ResNet18, with the betas set to (0.9, 0.98), a learning rate of 0.0001, and a batch size of 64, as this configuration provided an optimal balance between accuracy and computational efficiency.

### 3.3. Test Set Performance Evaluation

After selecting the optimal hyperparameter configuration, we conducted a comprehensive evaluation of our model on an independent test set comprising 194 samples. Given the inherent variability within the healthy population, we computed the MSE, MAE, and MRE for the predicted material parameters $C_{a}$ and $C_{b}$ to quantify the deviation from the ground truth.

For $C_{a}$, we performed a linear regression analysis comparing the ResNet18-predicted values with the corresponding FEM-derived reference values. This analysis yielded a regression function of:

(7) y=0.7987×x+0.0559,

with a correlation coefficient of approximately 0.9277. The model achieved an MSE of 0.0004, an MAE of 0.0146, and an MRE of about 4.97% for $C_{a}$, indicating a high level of accuracy and stability in its prediction.

Similarly, for $C_{b}$, the regression analysis produced a function of:

(8) y=0.8250×x+0.0502,

with a correlation coefficient of approximately 0.9258. The corresponding mean error metrics were an MSE of 0.0003, an MAE of 0.0139, and an MRE of around 5.00%.

**Figure 5:**
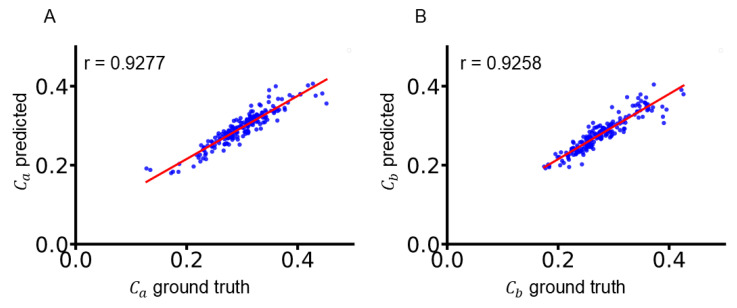
Scatterplots with regression lines for the predicted myocardial material parameters. (**A**) $C_{a}$. (**B**) $C_{b}$. Each panel displays the corresponding correlation coefficient r between the predicted values and the FEM-derived ground truth. The red lines represent the regression lines resulting from linear regression analysis comparing the ResNet18-predicted values with the corresponding FEM-derived reference values.

These results demonstrate that the proposed deep neural network can accurately predict the myocardial material parameters from routine imaging data, with strong agreement between the predicted values and the FEM-derived ground truth (Figure 5).

### 3.4. Performance Evaluation on the ACDC Pathological Dataset

To further assess our model’s performance under pathological conditions, we evaluated it on the publicly available ACDC dataset [22], focusing exclusively on pathological samples. Specifically, we analyzed 30 subjects diagnosed with abnormal right ventricle (ARV) and 30 subjects with hypertrophic cardiomyopathy (HCM). For the ARV group, the MREs were 11.99% for $C_{a}$ and 15.34% for $C_{b}$. In contrast, for the HCM group, the corresponding MREs were 19.10% for $C_{a}$ and 23.57% for $C_{b}$. These findings demonstrate that, in pathological conditions, the prediction accuracy decreases compared to that observed in healthy subjects, likely due to the increased heterogeneity in myocardial tissue properties. This observation provides an important reference for further refinement of parameter estimation in diseased hearts.

## 4. Discussion

In this study, we propose a deep learning-based approach for the rapid estimation of myocardial material parameters from routine CMRI data. The results demonstrate that deep learning can serve as an effective and efficient alternative to traditional inverse FE methods [23,24]. Compared to conventional approaches that require complex iterative optimization on high-performance computing systems with Abaqus—typically taking around 7 h—our method significantly reduces the computational cost while maintaining high accuracy, enabling predictions in under one second. This efficiency gain highlights the potential of deep learning in accelerating cardiac biomechanics analysis and broadening its clinical applications.

Analysis of the FEM-derived parameter distributions indicates that $C_{a}$ and $C_{b}$ exhibit relatively narrow ranges in the dataset, reflecting the homogeneity of the myocardial tissue properties in the studied population. Our hyperparameter tuning experiments indicate that the model performance is particularly sensitive to the learning rate and batch size, with optimal results achieved within a learning rate range of 1 × 10^−5^ to 1 × 10^−4^ and smaller batch sizes. In addition, the choice of network architecture plays a critical role in balancing prediction accuracy with computational efficiency. For example, our experiments with a ViT-based model yielded error metrics comparable to those of the CNN-based models; however, the significantly higher computational cost and training time (approximately four times that of ResNet18) rendered it less practical for our application. Ultimately, we selected ResNet18 as the final model because it offers robust performance with faster convergence and lower computational demands.

The test set evaluation confirms the strong predictive ability of the proposed model, particularly in estimating the $C_{a}$ with high accuracy and stability, while the $C_{b}$ predictions exhibit slightly higher errors. This discrepancy may arise due to the greater intrinsic variability in $C_{b}$, as it may be more sensitive to myocardial microstructural properties. These findings highlight the challenges associated with modeling myocardial material parameters, particularly the need for strategies that improve the estimation of $C_{b}$. Future work could explore integrating the biomechanical priors or leveraging multi-task learning approaches to better capture the variations in myocardial material parameters. Moreover, incorporating additional physiological constraints may enhance the model’s ability to infer myocardial stiffness more accurately.

One important consideration in our study is the use of mid-layer 2D images for the parameter estimation. While these mid-layer slices capture essential structural information of the left ventricle with high computational efficiency, we recognize that they do not fully represent the three-dimensional geometry of the heart. A 3D approach could provide a more comprehensive depiction of the cardiac structure, potentially leading to even more accurate parameter estimation. However, 3D data are typically much larger in volume, which not only increases the computational burden but also raises the risk of overfitting—especially given the potential anisotropy present in the data. Moreover, in clinical practice, cine MRI scans often exhibit variability in the slice thickness or *z*-axis spacing across different scanners, making high-resolution 3D reconstructions difficult to achieve consistently. Consequently, extracting representative mid-layer 2D slices from low-resolution scans offers a pragmatic and robust solution that is better suited to the complexities of real-world clinical settings. In future work, we plan to explore the integration of 3D data using advanced deep learning techniques, while implementing strategies to mitigate overfitting, to further enhance the robustness and accuracy of the myocardial parameter estimation.

Direct in vivo measurement of myocardial material parameters is highly challenging and typically invasive, which has resulted in a lack of large-scale, standardized datasets for direct measurement. Consequently, our study relies on FEM-derived parameters as a surrogate ground truth. FEM simulation methods, which have been extensively validated in both experimental and clinical studies, provide a feasible and reproducible means of estimating the myocardial material properties under current conditions. Although the use of FEM-derived parameters introduces certain uncertainties, it remains the most practical approach available at present. In future work, we plan to explore non-invasive measurement techniques, such as magnetic resonance elastography (MRE) [25,26], to further validate and refine our model’s predictions, thereby enhancing its clinical applicability.

Furthermore, it is important to highlight that the neural network in this study was trained exclusively on CMRI data from healthy subjects, which significantly impacts its clinical applicability. The myocardial tissue properties in healthy individuals tend to be relatively homogeneous, whereas pathological cardiac conditions are often accompanied by substantial tissue remodeling and altered mechanical properties. As a result, the model’s generalizability to pathological cases may be compromised. For instance, in diseases such as hypertrophic cardiomyopathy (HCM), the myocardial material parameters can exhibit greater inter-individual variability [27]. Since the proposed model has not been trained under these pathological conditions, its predictions may deviate from the actual values. This discrepancy in the data distribution constrains the model’s applicability in real-world clinical settings. Therefore, future research should focus on expanding the diversity of the training data by incorporating subjects with various cardiac pathologies. Additionally, strategies such as data augmentation or transfer learning could be explored to enhance the model’s robustness and generalizability in pathological conditions.

## 5. Conclusions

In conclusion, this study presents a deep learning-based framework for estimating the myocardial material parameters from routine CMRI data, providing a computationally efficient alternative to traditional inverse FE methods. The proposed ResNet18 model achieved high predictive accuracy, with mean relative errors below 5%, demonstrating strong generalizability across the dataset. This method provides a promising approach for efficient myocardial material characterization and lays the foundation for future applications in cardiac biomechanics and computational modeling.

## Figures and Tables

**Table 1:** Reference values of the eight parameters.

	$a_{0}$ (kPa)	$b_{0}$	$a_{f0}$ (kPa)	$b_{f0}$	$a_{s0}$ (kPa)	$b_{s0}$	$a_{fs0}$ (kPa)	$b_{fs0}$
Gao et al. [19]	0.180	2.600	3.340	2.730	0.690	1.110	0.310	2.580
Borowska et al. [18]	0.319	4.605	3.819	9.279	0.789	3.773	0.619	5.150

## Data Availability

Data can be obtained from the corresponding author upon reasonable request.
